# 

**Published:** 1874-04

**Authors:** 


### NEW PUBLICATIONS.

"The Animal Kingdom," published by the American Society for the Prevention of Cruelty to Animals, under the direction of Mr. Bergh, the great friend of the helpless brute creation, says of "*Baldwin's Monthly*," "that its editor, Baldwin the Clothier, finds time to send out fifty thousand copies of his journal, which is one of the neatest and most creditable papers published in New York City." Baldwin's sales of clothing on retail in six years amounted to nearly six million dollars. Such a result could not be found elsewhere on the globe. It is a legitimate consequence of a strict adherence to the simple rule, the money must be paid on delivery of the goods. If this practice of pay as you go were universal, almost half the anxieties of the human family would be prevented. Owe nobody; trust nobody.

### SCRIBNER'S MONTHLY MAGAZINE

for April comes to us laden, as usual, with good things. Among the articles of especial interest are "The Great South: A Ramble in Virginia," illustrated; "Christ's Resurrection Scientifically Considered;" "Concerning Some Imperial Booty;" "The Health and Physical Habits of English and American Women."

### HARPER BROTHERS' PUBLICATIONS.

**Scribner's Monthly Magazine,** $4 a year.

**Harpers' New Monthly,** now in its forty-eighth volume, largely illustrated, $4 a year.

**Harpers' Weekly,** $4 a year.

**Harpers' Bazar,** $4 a year.

**Atlantic Monthly,** $4 a year.

**Appleton's Journal,** $4 a year.

**Galaxy,** $4 a year.

**Blackwood's Magazine,** $4 a year.

We will furnish either of the above publications with **Hall's Journal of Health and Miscellany** at $4.50 a year.

Remit by Post-office Money Order, Registered Letter or Draft on New York, to

**E. H. GIBBS & CO.,**
**84 Broadway, New York.**

